# Effects of Vanillic Acid on Dynamic Fermentation Parameter, Nitrogen Distribution, Bacterial Community, and Enzymatic Hydrolysis of Stylo Silage

**DOI:** 10.3389/fmicb.2021.690801

**Published:** 2021-08-25

**Authors:** Liwen He, Sen Li, Cheng Wang, Xiaoyang Chen, Qing Zhang

**Affiliations:** ^1^State Key Laboratory of Animal Nutrition, College of Animal Science and Technology, China Agricultural University, Beijing, China; ^2^College of Forestry and Landscape Architecture, Guangdong Province Research Center of Woody Forage Engineering Technology, South China Agricultural University, Guangzhou, China; ^3^Guangdong Key Laboratory for Innovative Development and Utilization of Forest Plant Germplasm, South China Agricultural University, Guangzhou, China; ^4^State Key Laboratory for Conservation and Utilization of Subtropical Agro-Bioresources, South China Agricultural University, Guangzhou, China

**Keywords:** bacterial community, silage quality, enzymatic hydrolysis, protein preservation, vanillic acid

## Abstract

Vanillic acid (**VA**) is a phenolic acid derivative commonly found in plants and foods, with a pleasant creamy odor and pharmacologic activities, which is hypothesized to help improve silage fermentation. The silage profile of stylo silage ensiled with addition of VA was evaluated. The results showed that VA addition resulted in the decrease of pH value (5.22 vs. 4.33), dry matter loss (5.37 vs. 2.51% DM), and ammonia-N proportion (14.57 vs. 1.51% CP) of stylo silage as well as the increase of lactic acid concentration (0.51 vs. 1.17% DM), true protein proportion (51.18 vs. 58.47% CP), and saccharification yield (113.64 vs. 126.40 mg/g DM). Meanwhile, bacterial community of stylo silage was altered, where the relative abundance of *Enterobacter*, *Clostridium*, and *Kosakonia* decreased and that of *Commensalibacter* and *Methylobacterium* increased. In conclusion, it is suggested that VA could be used as a novel silage additive to improve silage fermentation and nutrient preservation of stylo silage.

## Introduction

Extensive proteolysis might be troublesome in silage production due to the inferior property of raw material, such as low water-soluble carbohydrate (**WSC**) content, high buffering capacity, or low lactic acid bacteria population. Measures have been taken to improve silage fermentation, where kinds of additives have been investigated to enhance nutrient preservation, digestibility, aerobic stability, or even functional activity (Muck et al., 2018). Nowadays, more attention has been paid to the issues of hygiene, safety, and environmental impact in silage production, so silage additives are expected to be multifunctional beyond improving nutrient preservation (Muck et al., 2018; Wilkinson and Rinne, 2018). Moreover, natural plant products with selected antimicrobial activity and other potential activities are attracting more attention.

Vanillic acid [4-hydroxy-3-methoxybenzoic acid (VA)], an oxidized form of vanillin, is a phenolic acid derivative with a pleasant creamy odor (Salau et al., 2020) and is commonly found in plants and foods like herbs, tea, and wine (Yao et al., 2020). It possesses a variety of pharmacologic activities such as inhibiting carcinogenesis, antioxidative, antihypertensive, anti-inflammatory, and antimicrobial activity (Calixto-Campos et al., 2015; Qian et al., 2020; Salau et al., 2020). Moreover, such biological functions might be partly owed to its inhibitory effects on different classes of enzymes like cyclooxygenase-2, phospholipases, and proteases (Henrique Cesar et al., 2020). Recently, VA has been widely used as preservatives, a flavoring agent in foods, cosmetics, and drugs, or an antioxidant with beneficial activities (Yao et al., 2020). Furthermore, it is reported that extracting solution of VA-rich *Angelica sinensis* and *Gardeniae Fructus* could promote lactic acid production and improve silage quality of alfalfa silage (Wang, 2012; Calixto-Campos et al., 2015; Salau et al., 2020). Accordingly, it is hypothesized that VA might be a promising silage additive due to its natural source, antimicrobial activity, protease inhibition, etc. Furthermore, relative to silage inoculant LAB, it is more convenient to preserve the functional activity of plant-source VA and its application condition is more robust. However, little information has been reported about the application of VA in silage fermentation so far.

Therefore, this study was conducted to investigate the effects of VA on silage fermentation. The fermentation parameter, nitrogen distribution, bacterial community, and enzymatic hydrolysis of stylo silage ensiled with addition of VA were analyzed on days 3, 7, 14, and 30 of ensiling fermentation.

## Materials and Methods

### Raw Material Preparation and Silage Processing

Fresh stylo (*CIAT* 184) was harvested on the trial field and then manually chopped into 1–2-cm length using a portable straw chopper (L-40, Shandong Yijia Hardware, China) in the lab. Immediately, the chopped forage was evenly mixed and ensiled without (**CK**) or with addition of 1.0 or 2.0% vanillic acid (**VA**) on a fresh matter (**FM**) basis, where VA was added in the form of powder. In total, 36 lab-level bagsilos (3 treatments × 4 sampling points × 3 replicates), 200 g raw material per silo, were individually prepared using polythene plastic bags (20 × 30 cm; maximum capacity of 300 g). Following recording the initial weight, all the prepared silos were kept in a carton and stored at room temperature (28–30°C). To monitor the dynamic changes of silage fermentation, three silage bags of each treatment were randomly unsealed for sample collection on days 3, 7, 14, and 30 of ensiling, which were analyzed for fermentation parameters, bacterial community, and nutrient preservation. Additionally, each bag was individually weighed again at sampling time, i.e., final weight, which would be used to evaluate dry matter loss (**DML**) with the help of initial weight and their corresponding dry matter (**DM**) content.

### Determination of Microbial Population, Fermentation Parameter, and Nitrogen Distribution

Bacteria solution was prepared by suspending silage samples in sterilized saline, and lactic acid bacteria (**LAB**), coliform bacteria, yeasts, and molds were separately cultivated with Man Rogosa Sharpe (MRS), Violet Red Bile, and Rose Bengal agar, and then enumerated after incubating at 30°C for 3–5 days. Another silage sample was soaked with distilled water at a ratio of 1:9 (*w*/*v*) for the analyses of pH, ammonia-nitrogen (**ammonia-N**), and organic acid (lactic acid, acetic acid, propionic acid, butyric acid), where pH was measured by a glass electrode pH meter (PHS-3C, INESA INSTRUMENT), ammonia-N content was determined in colorimetry (Broderick and Kang, 1980), and organic acid profile was analyzed using high-performance liquid chromatography (Agilent 1200). The remaining silage of each bag was oven-dried (65°C, 48 h) for DM determination as well as the analyses of nitrogen distribution (crude protein, true protein, and non-protein-N) referring to the method of Licitra et al. (1996). Additionally, the comprehensive quality of stylo silage was scored with the following equation (Flieg, 1938):

Flieg’sscore=220+(2×DM%-15)-40×pH.

### Saccharification Potential Evaluation Using Cellulase Hydrolysis Method

To evaluate the saccharification potential of the mature silage, enzymatic hydrolysis was performed using a cellulolytic enzyme mixture SAE0020 (Sigma; 120 FPU/ml) and a β-glucosidase preparation (Sigma; 30 CBU/mg) referring to He et al. (2019b). The incubation fluid was prepared with 50 mM sodium citrate buffer (pH 4.80), and the reaction was conducted in 125 ml Erlenmeyer flasks at a solid loading of 5% (w/v) and enzyme loadings of 20 FPU/g and 15 CBU/g substrate. Meanwhile, substrate blanks without enzyme and enzyme blanks without substrate were set in parallel with other samples. Additionally, proclin was added at the level of 0.02% (*v*/*v*) to prevent possible microorganism contamination. All the prepared flasks were incubated in a shaker setting at 50? and 180 rpm/min for 72 h, where the enzymatic hydrolysate was sampled at 12, 24, 48, and 72 h of incubation and centrifuged (6,000 × *g* for 5 min), and then WSC content was determined with the method of 3,5-dinitrosalicylic acid (MOA, 2015).

### Bacterial Community Analysis by 16S rDNA Sequencing Technique

The DNA extraction and sequencing analysis were carried out by the service of Gene Denovo Co., Ltd. (Guangzhou, China). In brief, bacterial DNA was extracted with DNA extraction kit (Omega Biotek, Norcross, GA, United States) and PCR amplification was conducted with the primers (341F: CCTACGGGNGGCWGCAG; 806R: GGACTACHVGGGTATCTAAT) targeting at the V3–V4 regions of 16S rDNA. A 50-μl reaction system containing 5 μl of 10 × KOD buffer, 5 μl of 2.5 mM dNTPs, 1.5 μl of each primer (5 μM), 1 μl of KOD polymerase, and 100 ng of template DNA was used. The reactions were conducted in triplicate, and the mixture of the amplicons was used to perform sequencing. Amplicons were purified using an AxyPrep DNA Gel Extraction Kit (Axygen Biosciences, Union City, CA, United States) and quantified using QuantiFluor-ST (Promega, United States). Following purification and quantification, the amplicons were sequenced on Illumina Hiseq 2500. Sequencing data were analyzed in steps as stated by Wang et al. (2019). Paired-end clean reads were merged using FLSAH (version 1.2.11) with a minimum overlap of 10 bp and mismatch error rate of 2%, and then noisy sequences were filtered with QIIME (version 1.9.1). Subsequently, chimera checking based on the reference database^1^ was performed using UCHIME algorithm.^2^ Operational taxonomic unit (OTU) with 97% identities was generated using UPARSE pipeline. Taxonomy assignment was performed using Ribosome Database Project (RDP) classifier (version 2.2). The metrics mainly containing Sobs, Shannon, Simpson, Chao, Ace, and Good_coverage were calculated in QIIME (version 2.15.3). Principal component analysis (PCA) was performed and plotted in R software (version 3.5.1). Functional gene prediction was conducted using Tax4Fun as reported in Xie et al. (2018). The sequencing data reported in this study was archived in the Sequence Read Archive (SRA) with the accession number SRP221902.

### Statistical Analysis

Statistical analyses were conducted using the PROC MIXED procedure of statistical software SAS 9.3 (SAS Institute Inc., Cary, NC, United States). Analysis of variance was conducted in a mixed model with treatment, sampling day, and the interaction of treatment by sampling day as fixed effects and silo as a random effect. The model was as follows:

$${\text{Y}}_{\text{ijk}}{\text{ = μ + Silo}}_{\text{i}}{\text{ + Trt}}_{\text{j}}{\text{ + Day}}_{\text{k}}{\text{ + Trt}}_{\text{j}}\;\times {\text{ Day}}_{\text{k}}{\text{ + e}}_{\text{ijk}},$$

where *Y*_ijk_ is the dependent variable, *μ* is the overall mean, Silo_i_ is the random effect of the *i*th silo, Trt_j_ is the effect of the *j*th treatment, Day_k_ is the effect of the *k*th sampling day, Trt_j_ × Day_k_ is the interaction effect of the *j*th treatment by the *k*th sampling day, and *e*_ijk_ is the residual error. For the repeated measures analysis, the covariance structure with unstructured type was used and silo was set as repeated subject. Results were reported as least squares means, and the PDIFF option was used to test for treatment differences among least squares means, along with the contrasts of Control and treatment, and 1 and 2% VA treatment. Significant difference was declared at the level of *p* < 0.05. Additionally, single-factor analysis of variance was conducted for the comparison of saccharification WSC yield. The sequencing analysis was performed on OmicShare platform.^3^

## Results

### General Characteristics of Fresh Stylo Used for Silage Production

In the present study, fresh stylo had a low DM content (23.94%) and a moderate crude protein content of 16.12% DM, where true protein proportion was high up to 90.95%. Its epiphytic LAB population was 4.41 log_10_ CFU/g FM, along with relatively high population of coliform bacteria (5.39 log_10_ CFU/g FM), yeasts (5.61 log_10_ CFU/g FM), and molds (4.53 log_10_ CFU/g FM).

### Fermentation Quality of Stylo Silage

Ensiling characteristics of stylo silage ensiled with addition of VA are summarized in Table 1. In the present study, DML of stylo silage (CK group) after 30-day fermentation was 5.37% DM and was reduced (*p* < 0.01) by the addition of VA, down to around 2.50% DM, but no difference (*p* > 0.05) was found between the two VA treatments. The addition of VA decreased (*p* < 0.01) the pH value (5.43, 4.63, 4.35) from the outset and showed dose effect (*p* < 0.01). It also led to the decrease (*p* < 0.01) of the populations of LAB (7.96, 5.45, and 4.34 log_10_ CFU/g FM) and coliform bacteria (6.09, 4.52, and 3.77 log_10_ CFU/g FM) as well as the increase (*p* < 0.01) of yeast number (2.00, 3.90, and 3.06 log_10_ CFU/g FM) along with significant dose effect (*p* < 0.01). Meanwhile, VA addition resulted in the increase (*p* < 0.01) of lactic acid (0.68, 1.20, and 1.19% DM) and propionic acid (0.55, 0.67, and 0.64% DM) concentrations but without dose effect (*p* > 0.05). However, acetic acid and butyric acid were only detected in the CK silage. Additionally, VA addition increased (*p* < 0.01) Flieg’s score of stylo silage (34.78, 70.50, 83.74) with 2% VA better (*p* < 0.01).

**TABLE 1:** Effect of vanillic acid on dynamic fermentation parameters of stylo silage.

**Item**	**Treatment**	**Days of ensiling**				**Mean**	**SEM**	***p*-Value**			**Contrast**	
		**D3**	**D7**	**D14**	**D30**			**Treatment**	**Day**	**Interaction**	**CK vs. VA**	**1 vs. 2%**
Dry matter (%)	CK	23.86	24.34	23.92	23.96	24.02	0.20	<0.01	<0.01	<0.01	<0.01	<0.01
	1%	24.93	25.58	25.54	26.55	25.65						
	2%	26.02	26.36	26.62	27.14	26.53						
Dry matter loss (% DM)	CK	0.88	1.16	2.57	5.37	2.50	0.05	<0.01	<0.01	<0.01	<0.01	0.61
	1%	0.39	0.52	0.93	2.51	1.09						
	2%	0.38	0.51	0.87	2.53	1.08						
pH	CK	5.55	5.52	5.55	5.20	5.46	1.73E-05	<0.01	0.01	0.02	<0.01	<0.01
	1%	4.72	4.57	4.71	4.58	4.65						
	2%	4.44	4.34	4.32	4.33	4.36						
Lactic acid (% DM)	CK	1.07	0.60	0.53	0.51	0.68	0.07	<0.01	0.01	<0.01	<0.01	0.87
	1%	1.10	1.23	1.29	1.17	1.20						
	2%	1.23	1.11	1.27	1.15	1.19						
Acetic acid (% DM)	CK	0.11	0.28	0.62	1.13	0.34	–	–	–	–	–	–
	1%	ND	ND	ND	ND	–						
	2%	ND	ND	ND	ND	–						
Propionic acid (% DM)	CK	0.42	0.51	0.62	0.65	0.55	0.07	0.01	0.32	0.04	0.02	0.77
	1%	0.85	0.62	0.61	0.61	0.67						
	2%	0.75	0.63	0.54	0.63	0.64						
Butyric acid (% DM)	CK	ND	ND	0.46	0.99	–	–	–	–	–	–	–
	1%	ND	ND	ND	ND	–						
	2%	ND	ND	ND	ND	–						
LAB (log_10_ CFU/FM)	CK	8.27	8.43	7.98	7.17	7.96	0.21	<0.01	0.03	<0.01	<0.01	<0.01
	1%	5.15	5.16	5.77	5.71	5.45						
	2%	4.00	4.54	4.39	4.42	4.34						
Coliform (log_10_ CFU/FM)	CK	7.86	7.45	6.03	3.00	6.09	0.39	<0.01	<0.01	<0.01	<0.01	0.07
	1%	4.62	4.91	4.31	4.25	4.52						
	2%	4.00	3.42	3.91	3.74	3.77						
Yeast (log_10_ CFU/FM)	CK	2.00	2.00	2.00	2.00	2.00	0.33	<0.01	<0.01	<0.01	<0.01	<0.01
	1%	3.86	3.50	4.12	4.13	3.90						
	2%	2.00	3.43	2.43	4.39	3.06						
Mold (log_10_CFU/FM)	CK	3.23	<2.00	<2.00	<2.00	–	0.15	–	–	–	–	–
	1%	2.43	2.26	<2.00	<2.00	–						
	2%	2.00	2.33	<2.00	<2.00	–						
Flieg’s score^a^	CK	30.58	32.75	30.85	44.92	34.78	1.62	<0.01	<0.01	<0.01	<0.01	<0.01
	1%	66.20	73.36	67.67	74.76	70.50						
	2%	79.56	83.99	85.31	86.08	83.74						

*CK, blank control; 1%, addition of 1% vanillic acid; 2%, addition of 2% vanillic acid; D3, sampling on day 3 of ensiling fermentation; D7, sampling on day 7 of ensiling fermentation; D14, sampling on day 14 of ensiling fermentation; D30, sampling on day 30 of ensiling fermentation; CK vs. VA, comparison of CK group and VA-treated groups;1% vs. 2%, comparison of 1% VA- and 2% VA-treated groups; VA, vanillic acid, SEM, standard error of means; ND, not detected, “–” default; DM, dry matter; FM, fresh matter.*

*^a^Flieg’s score = 220 + (2 × DM% – 15) – 40 × pH.*

### Nitrogen Distribution and Enzymatic Saccharification of Stylo Silage

The dynamic changes of nitrogen distribution are presented in Table 2. In the present study, the content and proportion of true protein decreased (*p* < 0.01) during ensiling fermentation, and those of non-protein-N and ammonia-N increased (*p* < 0.01). In comparison, VA addition led to the increase (*p* < 0.05) of true protein proportion (56.91, 61.99, 62.12%) as well as the decrease (*p* < 0.01) of non-protein-N (6.58, 5.46, and 5.58% DM) and ammonia-N (0.19, 0.02, and 0.02% DM) content or proportion without dose effect (*p* > 0.05).

**TABLE 2:** Effect of vanillic acid on dynamic nitrogen distribution of stylo silage.

**Item**	**Treatment**	**Days of ensiling**				**Mean**	**SEM**	***p*-Value**			**Contrast**	
		**3**	**7**	**14**	**30**			**Treatment**	**Day**	**Interaction**	**CK vs. VA**	**1 vs. 2%**
Crude protein (% DM)	CK	15.18	15.77	15.43	14.70	15.27	0.32	0.03	< 0.01	0.11	0.06	0.92
	1%	15.00	14.27	14.29	14.07	14.41						
	2%	14.88	15.12	14.43	14.24	14.43						
True protein (% DM)	CK	9.89	8.87	8.62	7.38	8.69	0.39	0.12	< 0.01	0.02	0.16	0.53
	1%	10.41	8.93	8.41	8.02	8.94						
	2%	10.00	8.47	9.36	8.33	9.04						
Non-protein-N (% DM)	CK	5.29	6.89	6.82	7.07	6.58	0.50	0.01	< 0.01	0.13	0.03	0.67
	1%	4.59	5.34	5.87	6.04	5.46						
	2%	4.55	6.64	4.65	5.91	5.58						
Ammonia-N (% DM)	CK	0.05	0.14	0.22	0.34	0.19	0.004	< 0.01	<0.01	< 0.01	<0.01	0.47
	1%	0.01	0.01	0.02	0.04	0.02						
	2%	0.01	0.01	0.02	0.03	0.02						
TP/CP (%)	CK	65.16	56.29	55.92	51.18	56.91	2.86	0.02	< 0.01	0.02	0.04	0.93
	1%	69.40	62.60	58.91	57.05	61.99						
	2%	68.73	56.23	66.92	58.47	62.12						
NH_3_-N/CP (%)	CK	2.22	5.38	9.11	14.57	7.82	0.13	< 0.01	<0.01	< 0.01	<0.01	0.38
	1%	0.45	0.60	1.02	1.72	0.95						
	2%	0.43	0.53	0.90	1.51	0.83						

*CK, blank control; 1%, addition of 1% vanillic acid; 2%, addition of 2% vanillic acid; D3, sampling on day 3 of ensiling fermentation; D7, sampling on day 7 of ensiling fermentation; D14, sampling on day 14 of ensiling fermentation; D30, sampling on day 30 of ensiling fermentation; CK vs. VA, comparison of CK group and VA-treated groups; 1% vs. 2%, comparison of 1% VA- and 2% VA-treated groups; VA, vanillic acid; SEM, standard error of means; DM, dry matter; TP/CP, the proportion of true protein in crude protein; NH_3_-N, ammonia-N (expressed in CP equivalent); NH_3_-N/CP, the proportion of ammonia-N in crude protein.*

**FIGURE 1:**
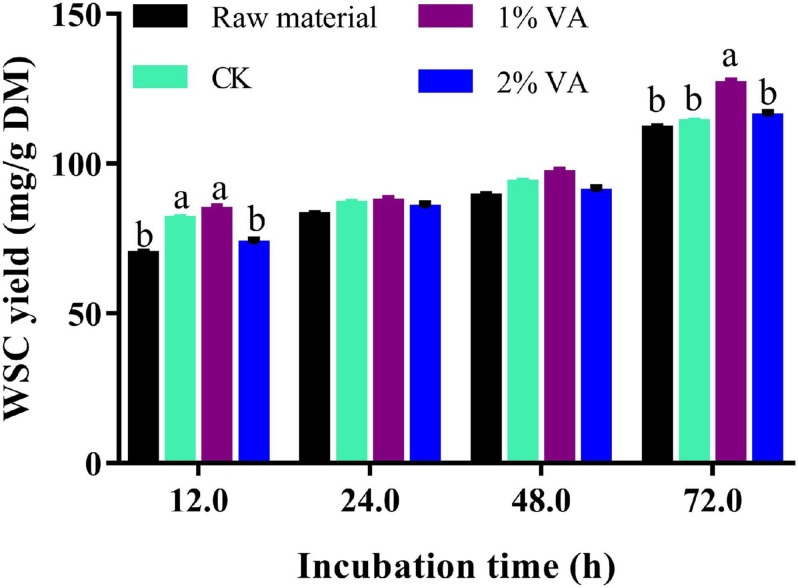
Enzymatic water-soluble carbohydrate (WSC) yield of mature stylo silage (30 days of ensiling) ensiled with the addition of vanillic acid [CK: blank control, 1 and 2%: addition of 1 or 2% vanillic acid. olumns with different letters (a and b) at one time point differ at *p* < 0.05].

As illustrated in Figure 1, enzymatic WSC yield of stylo silage (30 days ensiling fermentation) was not less than that of raw material. Moreover, the addition of 1% VA led to increased WSC yield of 72 h incubation but not for 2% VA treatment.

### Bacterial Community of Stylo Silage

The values of Goods_coverage were over 0.99, and the alpha diversity indices including Sobs, Shannon, Simpson, Chao, and Ace were all higher in stylo silage relative to fresh stylo (Table 3). Compared with the control silage, VA addition resulted in the decrease (*p* < 0.01) of Shannon and Simpson as well as the increase (*p* < 0.01) of Sobs without dose effect (*p* > 0.05; Table 3).

**TABLE 3:** Effect of vanillic acid on bacterial alpha diversity of stylo silage.

**Item**	**Treatment**	**Days of ensiling**				**Mean**	**SEM**	***p*-Value**			**Contrast**	
		**3**	**7**	**14**	**30**			**Treatment**	**Day**	**Interaction**	**CK vs. VA**	**1 vs. 2%**
Sobs	CK	1,578.3	1,444.0	1,498.7	1,374.7	1,473.9	55.2	< 0.01	<0.01	< 0.01	<0.01	0.08
	1%	1,685.3	1,716.0	1,642.7	1,693.0	1,684.3						
	2%	1,542.0	1,634.0	1,762.0	1,487.7	1,606.4						
Shannon	CK	6.08	6.01	6.15	5.77	6.00	0.20	< 0.01	<0.01	0.03	< 0.01	0.63
	1%	4.70	5.42	5.82	5.69	5.40						
	2%	4.93	5.20	5.90	5.13	5.29						
Simpson	CK	0.96	0.96	0.96	0.94	0.95	0.02	< 0.01	<0.01	< 0.01	<0.01	0.56
	1%	0.73	0.83	0.90	0.90	0.84						
	2%	0.83	0.81	0.86	0.91	0.85						
Chao	CK	2,468.4	2,356.6	2,380.7	2,142.2	2,337.0	87.6	0.06	0.01	0.03	0.75	0.09
	1%	2,420.3	2,476.6	2,514.0	2,481.5	2,473.1						
	2%	2,165.1	2,307.4	2,558.8	2,244.2	2,318.9						
Ace	CK	2,523.8	2,373.1	2,464.0	2,227.6	2,397.1	73.8	0.04	< 0.01	0.03	0.22	0.05
	1%	2,390.8	2,452.5	2,529.0	2,455.8	2,457.0						
	2%	2,133.9	2,316.2	2,500.3	2,190.7	2,285.3						
Goods_coverage	CK	0.9912	0.9920	0.9914	0.9923	0.9917	0.0004	0.27	0.02	0.20	0.98	0.50
	1%	0.9907	0.9912	0.9908	0.9920	0.9912						
	2%	0.9914	0.9905	0.9913	0.9924	0.9914						

*CK, blank control; 1%, addition of 1% vanillic acid; 2%, addition of 2% vanillic acid; D3, sampling on day 3 of ensiling fermentation; D7, sampling on day 7 of ensiling fermentation; D14, sampling on day 14 of ensiling fermentation; D30, sampling on day 30 of ensiling fermentation; CK vs. VA, comparison of CK group and VA-treated groups; 1% vs. 2%, comparison of 1% VA- and 2% VA-treated groups; VA, vanillic acid; SEM, standard error of means.*

**FIGURE 2:**
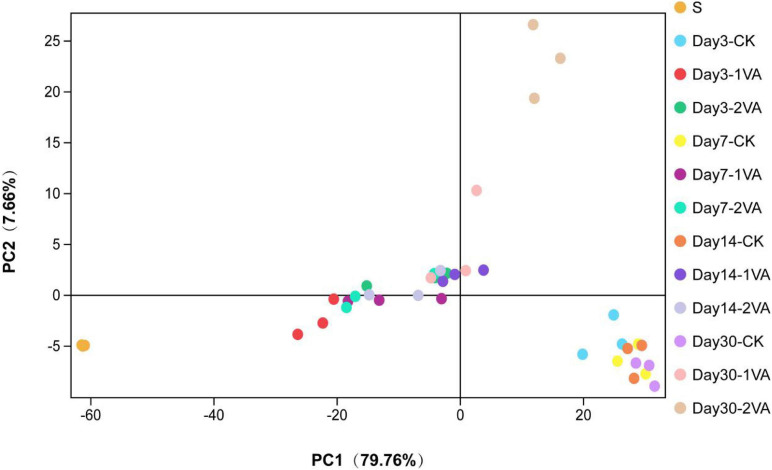
Principal component analysis of bacterial community in stylo silage ensiled with the addition of vanillic acid (S: raw material, i.e., fresh stylo, CK: blank control, 1 and 2%: addition of 1 or 2% vanillic acid).

PCA showed that, samples of the raw material, CK group, and VA groups were separately clustered, and the separation of the samples in each treatment became more apparent as ensiling fermentation went on Figure 2.

**FIGURE 3:**
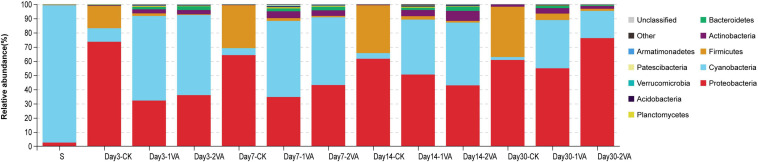
Relative abundance of bacterial community on phylum level in stylo silage ensiled with the addition of vanillic acid (S: raw material, i.e., fresh stylo, CK: blank control, 1 and 2%: addition of 1 or 2% vanillic acid).

The relative abundance on the phylum level of bacterial community in stylo silage is illustrated in Figure 3. In general, *Cyanobacteria* was the dominant phylum with the abundance of 96.66% in fresh stylo along with a low proportion (2.57%) of *Proteobacteria*, while *Proteobacteria* (60.75–73.57%) and *Firmicutes* (15.73–35.42%) became the main phyla in the CK silage, and *Cyanobacteria* (19.18–59.54%) and *Proteobacteria* (32.17–76.16%) were the two major phyla in the VA-treated silages. The relative abundance of *Cyanobacteria* (9.53–2.07%) and *Proteobacteria* (73.57–60.75%) decreased and that of *Firmicutes* (15.73–35.42%) increased in the CK silage, and the relative abundance of *Cyanobacteria* (59.54–19.18%) also decreased in the VA-treated silage and that of *Proteobacteria* (32.17–76.16%) increased. In comparison, VA application led to the decrease of *Firmicutes* and *Proteobacteria* as well as the increase of *Cyanobacteria.* Moreover, the phyla like *Actinobacteria* and *Bacteroidetes* were more abundant in the VA-treated silages.

**FIGURE 4:**
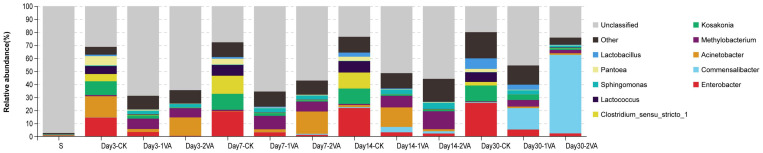
Relative abundance of bacterial community on genus level in stylo silage ensiled with the addition of vanillic acid (S: raw material, i.e., fresh stylo, CK: blank control, 1 and 2%: addition of 1 or 2% vanillic acid).

The relative abundance of bacterial community in stylo silage was also different on the genus level among raw material, CK silage and VA-treated silages, and a large proportion of the community was unclassified using the second-generation 16S rDNA sequencing analysis (Figure 4). In comparison, the proportion of classified bacteria was higher in the CK silage (68.73–80.06%) relative to the VA-treated silages (31.19–75.84%), and the classified proportion increased in the prolonged silage. When focusing on the classified bacteria, most of the stylo silage samples were jointly dominated by several genera. In detail, CK silage on day 3 of ensiling was dominated by *Acinetobacter* (16.2%), *Enterobacter* (14.45%), *Kosakonia* (10.06%), *Lactococcus* (6.10%), *Pantoea* (7.10%), and *Clostridium* (5.50%), the last five of which were the main genera through ensiling process. *Enterobacter*, *Acinetobacter*, *Sphingomonas*, *Methylobacterium*, and *Kosakonia* were the top five genera in the 1% VA-treated silage, while *Enterobacter*, *Commensalibacter*, *Acinetobacter*, *Sphingomonas*, and *Methylobacterium* were the main genera in the 2% VA-treated silage.

**FIGURE 5:**
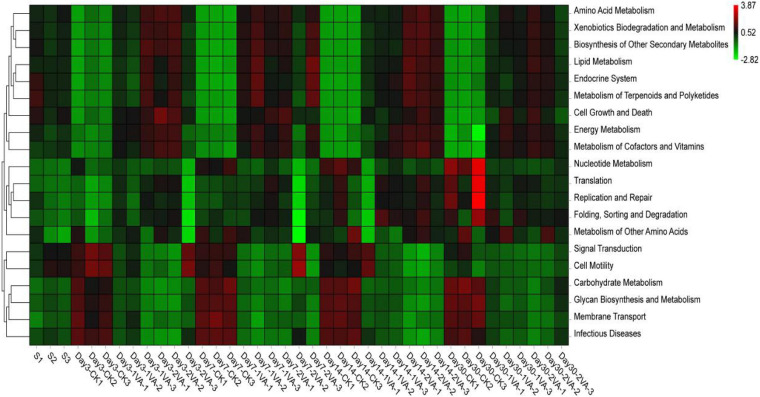
Heat map of 16S rRNA gene-predicted functional profiles of stylo silage ensiled with the addition of vanillic acid (S: raw material, i.e., fresh stylo, CK: blank control, 1 and 2%: addition of 1 or 2% vanillic acid).

16S rRNA gene-predicted functional profile (Figure 5) also showed that VA application altered lots of functional metabolism routes, such as upgrading the metabolism of amino acid, lipid, energy, terpenoids and polyketides, xenobiotics biodegradation, biosynthesis of other secondary metabolites, and downregulating the metabolism of carbohydrate, glycan biosynthesis, signal transduction, cell motility, membrane transport, and infectious diseases.

## Discussion

### General Characteristics of Fresh Stylo Used for Silage Production

Stylo is one of the most common forage in southern China with high biomass yield, being a quality protein source for ruminants. In the present study, the protein content of fresh stylo was a little higher than the reported data (He et al., 2019a, 2020). It might be due to the earlier mowing stage and better growth vigor of raw stylo. Meanwhile, its high true protein proportion proved it a quality protein source in consideration that true protein is generally higher in utilization efficiency relative to non-protein-N, especially nitrate-N or ammonia-N (Mcdonald et al., 1991). However, its high moisture content and low epiphytic LAB population along with the relatively high populations of coliform bacteria, yeasts, and molds, might not be beneficial to the dominance establishment of LAB or a rapid pH decline during ensiling process. Stylo silage without further treatments might bear a high risk of remarkable nutrient loss and extensive proteolysis in that a high moisture condition would favor clostridial fermentation and butyric acid generation (Borreani et al., 2018; He et al., 2019c). Accordingly, VA addition might help improve microbial fermentation and protein preservation during ensiling process due to its great antimicrobial activity and inhibitory effects on different classes of enzymes (Henrique Cesar et al., 2020; Yao et al., 2020).

### Fermentation Quality of Stylo Silage

Ensiling is an economical way to preserve moist forage with the help of LAB converting WSC into organic acid (mainly lactic acid) whereby lowering silage pH to inhibit the activity of spoilage organisms (Mcdonald et al., 1991). Even so, the respiration of plant cell and the activities of aerobic or anaerobic microorganisms would inevitably cause nutrient loss, and any effort inhibiting such activities would help to improve nutrient preservation (He et al., 2019d). In the present study, DML of stylo silage was reduced by the addition of VA, but no difference was found between the two VA treatments, indicating that VA application could improve nutrient preservation of stylo silage and the dose of 1% FM might be high enough.

Silage pH is mainly dictated by the generation of lactate and its intrinsic buffering capacity (Kung et al., 2018). Generally, pH 4.20 is regarded as a benchmark for quality silage, and pH 4.30–5.00 is common for acceptable legume silage due to its low sugar content and high buffering capacity (Mcdonald et al., 1991; Kung et al., 2018). In the present study, pH value of stylo silage was relatively high (pH 5.55), which was decreased by the addition of VA (pH 4.33). Regardless of other potential effects, VA addition would necessarily lower pH more or less due to its acidity property. Meanwhile, VA addition led to the decrease of LAB and coliform bacteria populations along with the increase of yeast number. It is documented that LAB can actively grow between the pH values of 4.50–7.50 (Hutkins and Nannen, 1993), and most undesirable bacteria are usually inhibited at pH < 4.50 but many yeast are capable to grow at pH 3.50 (Kung et al., 2003). It might be interpreted as the pH condition was low enough to inhibit the proliferation of LAB, coliform bacteria, and molds but not yeasts. Moreover, antifungal acetic acid was not detected in VA-treated silages, and VA might be used as a substrate for some fungi (Buswell et al., 1982; Kurek and Greń, 2014), partly explaining why yeast population increased in the VA-added silages. Noteworthily, yeasts are regarded as the culprit of silage spoilage under aerobic condition (Muck, 2010; He et al., 2019c), so VA application might discount aerobic stability of stylo silage.

In the present study, lactic acid concentration declined, and acetic acid and butyric acid accumulated in the CK silage, inferring that lactic acid was partially converted into acetic acid and butyric acid during ensiling fermentation. Such a metabolic transformation would inevitably lead to CO_2_ generation and energy loss (Mcdonald et al., 1991), which might partly account for the higher DML in the CK silage. Additionally, high butyric acid concentration (0.99% DM on day 30 of ensiling fermentation) is undesired in that butyric acid > 0.5% DM in ration would result in reduced feed intake and other metabolic diseases (Muck, 2010). Flieg’s evaluation system is a common method to evaluate silage quality based on the data of DM and pH (Flieg, 1938). According to the criterion of Flieg’s scoring system, the CK silage was classified as poor quality, while 1- or 2% VA-treated silage should be judged into the grade of good quality or very good quality. Accordingly, VA application did improve the fermentation quality of stylo silage.

### Nitrogen Distribution and Enzymatic Saccharification of Stylo Silage

Extensive proteolysis is a common issue in legume silage primarily due to its abundant intrinsic proteases and relative high pH condition (Li et al., 2018). Plant enzymes firstly hydrolyze protein into peptides and free amino acids, which are further degraded mainly by the deamination activity of undesirable bacteria like *Clostridium* and *Enterobacter*, generating amides, amines, ammonia, and ketonic acids (Kung et al., 2018). Thus, monitoring the concentration of non-protein-N or ammonia-N would be an easy way to reflect the extent of proteolysis. For ruminants, non-protein-N, especially ammonia-N, are less efficient in nitrogen retention than true protein, thus extensive proteolysis would lead to inferior nutritional value of silage and more nitrogen excretion in animal production (Mcdonald et al., 1991; He et al., 2020). In the present study, ammonia-N content or proportion was decreased by the addition of VA, indicating that the deamination activity of undesirable bacteria was inhibited. Meanwhile, non-protein-N content was lower in VA-treated silages along with a higher proportion of true protein. It is confirmed that the addition of VA inhibited proteolysis in stylo silage. As aforementioned, VA possesses the characters of acidity, antibacterial activity, and enzyme inhibition, thus it might exert an effect *via* inhibiting protease activity and microbial deamination activity.

Enzymatic hydrolysis is a simple way to evaluate the utilization efficiency of the biomass in biofuel production and microbial fermentation (He et al., 2019b). In the present study, enzymatic WSC yield of stylo silage was not less than that of raw material, indicating that ensiling preservation would not lower the utilization efficiency of the carbohydrates. Moreover, ensiling stylo with addition of 1% VA increased its enzymatic WSC yield. It might be interpreted that the addition of VA improved silage fermentation and nutrient preservation, including the digestible carbohydrates. Furthermore, storage in a slight acidic condition might also promote the digestion of cell wall components. Noteworthily, high VA residue in the silage might discount the activity of cellulase hydrolysis that is why 2% VA treatment did not increase the utilization potential of stylo silage. From the above, VA application improved nutrient preservation in stylo silage, including protein and digestible carbohydrates.

### Bacterial Community of Stylo Silage

Essentially, ensiling fermentation is a process of microbial competition, and the improvement of silage quality primarily results from the corresponding alteration of microbial community (Yang and Wang, 2018; He et al., 2020). Accordingly, sequencing the bacterial community might help us to illustrate the mechanism of VA improving silage quality. In the present study, the values of Goods_coverage > 0.99 indicated that the sequencing data were large enough to represent the actual profile of bacterial community in the silage. The alpha diversity indices were all higher in stylo silage relative to fresh stylo, indicating that the richness and diversity of bacterial community were increased in ensiling fermentation. In comparison, VA application resulted in the decrease of Shannon and Simpson and the increase of Sobs, suggesting that VA would lower bacterial community diversity of stylo silage, likely due to its antimicrobial activity. Furthermore, PCA analysis illustrated that samples of the raw material, CK group, and VA groups were separately clustered, and the separation in each treatment became more apparent as ensiling fermentation went on, suggesting that ensiling fermentation or VA application exerted a remarkable effect on the bacterial community. In short, the diversity analysis revealed that VA application did alter the bacterial community of stylo silage. Thus, it is believed that VA improved silage fermentation and nutrient preservation likely *via* optimizing microbial community.

On the phylum level of bacterial community, *Cyanobacteria* was the dominant phylum in fresh stylo along with a low proportion of *Proteobacteria*, while *Proteobacteria* and *Firmicutes* became the main phyla in the CK silage, and *Cyanobacteria* and *Proteobacteria* were the two major phyla in the VA-treated silages. Similarly, previous studies also reported that *Cyanobacteria* was the dominant phylumin stylo silage or in fresh tropical forages, and the bacterial community was altered by the addition of silage additives (He et al., 2019a; Li et al., 2019). *Cyanobacteria* is a photosynthesizing phylum of bacteria found in diverse environments, and their growth is affected by the factors of light, nutrient supply, and temperature (Carr and Whitton, 1982; Heberline, 2017). Referring to the dynamic changes of silage fermentation, the relative abundance of *Cyanobacteria* and *Proteobacteria* decreased and that of *Firmicutes* increased in the CK silage, and the relative abundance of *Cyanobacteria* also decreased in the VA-treated silages but that of *Proteobacteria* increased. In comparison, VA application led to the decrease of *Firmicutes* and *Proteobacteria* as well as the increase of *Cyanobacteria.* Moreover, the phyla like *Actinobacteria* and *Bacteroidetes* were more abundant in the VA-treated silages. It is indicated that the bacterial community of stylo silage was dramatically changed by the addition of VA.

Furthermore, the relative abundance of bacterial community in stylo silage was also different on the genus level among raw material, CK silage, and VA-treated silages, and a large proportion of bacterial community was unclassified using the second-generation 16S rDNA sequencing technique. It might be due to the relatively poor development of Cyanobacterial taxonomy, where most of Cyanobacteria cannot be cultured in the present knowledge (Palinska and Surosz, 2014). When focusing on the community of classified bacteria, CK silage on day 3 of ensiling was dominated by *Acinetobacter*, *Enterobacter*, *Kosakonia*, *Lactococcus*, *Pantoea*, and *Clostridium*, where the last five were the main genera during ensiling process. *Enterobacter*, *Acinetobacter*, *Sphingomonas*, *Methylobacterium*, and *Kosakonia* were the top 5 genera in the 1%VA-treated silage, while *Enterobacter*, *Commensalibacter*, *Acinetobacter*, *Sphingomonas*, and *Methylobacterium* were the main genera in the 2% VA-treated silage. Such a difference of bacterial community would mainly account for the improvement of silage quality.

Generally speaking, *Enterobacter* and *Clostridium* are undesirable bacteria in silage fermentation in that their activities would cause much protein degradation, dry matter loss, and ammonia and butyric acid production, hindering pH decline (Pahlow et al., 2003; Muck, 2010). Furthermore, feeding dairy cows with the silage abundant in *Clostridium* would likely lead to the decrease of feed intake and a higher ketosis incidence (Muck, 2010). *Kosakonia* and *Pantoea* are two new genera recently classified from the genus *Enterobacter* (Li, 2016). The decline of these bacteria in the VA-treated silages might contribute to the reduction of DML and ammonia-N content. Meanwhile, the higher abundance of *Lactococcus* and *Lactobacillus* in the CK silage might account for its more acid production given that they are common lactic acid bacteria in silage (Pahlow et al., 2003). Additionally, the abundance of *Methylobacterium* and *Sphingomonas* in stylo silage was increased by the addition of VA. Similarly, one of our previous studies showed that the addition of gallic acid led to the increase of *Methylobacterium* (He et al., 2019a). However, *Methylobacterium* are aerobic, neutrophilic, and facultative methylotrophic bacteria commonly found in plants (Doronina et al., 2002), and their abundance is reported to positively correlate with silage pH (Ogunade et al., 2018). *Sphingomonas* are Gram-negative aerobic *Alphaproteobacteria* commonly detected in agricultural by-product silage and considered to cause hydrolysis of soluble protein (Zhou et al., 2019). Thus, the increase of their abundance in silage might be undesirable but their exact roles in silage fermentation still need further research.

In addition, 16S rRNA gene-predicted functional profile also showed that VA application altered lots of the functional metabolism routes, such as upgrading the metabolism of amino acid, lipid, energy, xenobiotics biodegradation, and downregulating the metabolism of glycan biosynthesis and infectious diseases. It is inferred that the addition of VA might provoke the activity of bacterial biodegradation to clear xenobiotics, which might need to mobilize amino acid and energy-associated metabolism routes, and inhibit the proliferation of bacteria, especially the infectious bacteria. Such functional changes of bacterial community might explain the mechanism of VA antimicrobial activity.

## Conclusion

The present results showed that pH value, dry matter loss, butyric acid, and ammonia-N contents of stylo silage were decreased by the addition of VA at ensiling time, and lactic acid concentration, true protein proportion, and enzymatic WSC yield were increased. Meanwhile, bacterial community of stylo silage was altered, where the relative abundance of *Enterobacter*, *Clostridium*, and *Kosakonia* was decreased, and that of *Commensalibacter* and *Methylobacterium* was increased. It is suggested that VA could be used as a novel silage additive to improve silage quality of stylo silage, with the proper dose of 1% FM.

## Data Availability Statement

The datasets presented in this study can be found in online repositories. The names of the repository/repositories and accession number(s) can be found below: NCBI BioProject, PRJNA734254.

## Author Contributions

LH: conceptualization, methodology, writing, reviewing, and editing. SL: resources, methodology, and investigation. CW: methodology, investigation, data collection, and formal analysis. XC: validation and supervision. QZ: conceptualization, methodology, and visualization.

## Conflict of Interest

The authors declare that the research was conducted in the absence of any commercial or financial relationships that could be construed as a potential conflict of interest.

## Publisher’s Note

All claims expressed in this article are solely those of the authors and do not necessarily represent those of their affiliated organizations, or those of the publisher, the editors and the reviewers. Any product that may be evaluated in this article, or claim that may be made by its manufacturer, is not guaranteed or endorsed by the publisher.

## Footnotes

1
http://drive5.com/uchime/uchime_download.html

2
http://www.drive5.com/usearch/manual/uchime_algo.html

3
http://www.omicshare.com/tools
